# Circadian rhythms and the use of transfer learning for critically endangered crested argus *Rheinardia ocellata* in the Central Highlands of Vietnam: the implications for conservation

**DOI:** 10.1098/rstb.2024.0056

**Published:** 2025-06-12

**Authors:** Thanh Nguyen Chi, Thinh Tien Vu, Hoa Thi Nguyen, Dena Jane Clink

**Affiliations:** ^1^ Faculty of Forestry, Bac Giang Agro-Forestry University, Bich Dong, Viet Yen, Bac Giang, Vietnam; ^2^ Department of Wildlife, Vietnam National University of Forestry, Chuong My, Vietnam; ^3^ Institute for Tropical Biodiversity and Forestry, Xuan Mai, Chuong My, Ha Noi, Vietnam; ^4^ K. Lisa Yang Center for Conservation Bioacoustics, Cornell Lab of Ornithology, Cornell University, Ithaca, NY, USA

**Keywords:** circadian rhythms of vocal, crested argus, Kon Chu Rang, wildlife monitoring

## Abstract

Understanding the circadian rhythm of the calling behaviour of wild animals can guide efforts to monitor and conserve rare and endangered species using sound. Here, we use passive acoustic monitoring to investigate the vocal behaviour of the crested argus (*Rheinardia ocellata*) in Kon Chu Rang Nature Reserve, Gia Lai Province, Vietnam. We had three main objectives: (i) to investigate the performance of BirdNET transfer learning for automated detection of crested argus calls; (ii) to investigate the environmental predictors of crested argus calling; and (iii) to qualitatively investigate seasonal patterns of calling. We recorded continuously for 4–5 days at 40 recording points in 2021, and at 30 points in 2023. We also recorded the calls of crested argus at four fixed points from 2022 to 2023 to explore patterns of seasonal variation. For automated detection, we found acceptable performance with only 30 high-quality training samples (F1 score = 0.70). Our top model for calling during the 24 h period only included the time category, and we found that there was peak calling activity at dawn and dusk. We found peak calling activity during March and April. Our findings can contribute to planning effective monitoring of the critically endangered crested argus.

This article is part of the theme issue ‘Acoustic monitoring for tropical ecology and conservation’.

## 1. Introduction

Passive acoustic monitoring (PAM) involves deploying autonomous acoustic sensors or recording devices to capture sounds produced by vocal animals, natural events or anthropogenic activities. By studying these sounds, researchers can identify specific species, monitor biodiversity, study animal behaviour and assess environmental changes without the need for human observers [1]. PAM is a valuable tool for monitoring species that use sound for communication, particularly avian species. It offers significant advantages over visual surveys for studying loud yet elusive species that are rare and difficult to observe visually, as these species can be identified over long distances using sound. Autonomous recording devices have been shown to be more effective than traditional human-based surveys in environments with limited visibility or in regions with a high species diversity but few experienced field researchers, such as tropical areas [2–4]. Additionally, the logistics of conducting surveys in remote locations are simplified by using autonomous recorders, which are easier to install and more cost-effective compared with organizing travel and accommodation for human survey teams. This is particularly true for studies that cover large spatial and temporal scales.

Identifying the signals of interest in long-term acoustic recordings remains one of the major bottlenecks for the analysis of PAM data. Signals of interest may be detected by listening to the recordings or by visually scanning spectrograms. Manual annotation of spectrograms, aided by computer software such as Raven Pro 1.6 (K. Lisa Yang Center for Conservation Bioacoustics) or Avisoft-SASLab Pro (Avisoft), was the traditional way to analyse long-term recordings. However, for large datasets, visual scanning is time- and cost-prohibitive. The use of automated detection/classification algorithms implemented with existing packages in programming languages such as R or Python can allow for faster detection of the target sounds [5–8]. Recent advances in deep learning have revolutionized automated detection in bioacoustics applications [9]. Automated detection of bird sounds has been particularly fruitful, with algorithms such as BirdNET [10] and Perch [11] effectively classifying the calls of thousands of bird species. As training deep learning algorithms requires a substantial amount of training data, these algorithms leverage the use of open datasets such as those found on Xeno-Canto (https://xeno-canto.org/), wherein citizen scientists have contributed vast amounts of labelled training data.

For cases where large amounts of training data are not available, or access to high-power computers is limited, transfer learning can be useful. Transfer learning utilizes feature embeddings from trained machine learning models for a new task, often in a different domain from the original training data [12]. For example, transfer learning with convolutional neural networks trained on the ‘ImageNet’ dataset [13] was used successfully for automated detection of bioacoustic signals [14]. The ‘VGGish’ model, which was trained on the ‘AudioSet’ dataset, comprised labelled acoustic events from YouTube [15], has also been applied to bioacoustic applications [16]. Recently, the use of embeddings from global birdsong models like BirdNET and Perch has been shown to have superior performance over other transfer learning approaches [17]. Importantly, the use of embeddings can facilitate transfer learning in cases whre a limited number of training samples are available to train an effective automated detector [18]. For rare or understudied vocal species, researchers often have a limited number of high-quality training samples, which means approaches that work effectively with a small number of training samples are required. Traditional non-deep learning approaches, such as energy or template detectors [19], can also be used when a small number of training samples are available. Data augmentation can also help when the number of available training samples is low, with some commonly used bioacoustics augmentations including time-shifting, adding background noise and synthesis of new sounds [20].

Animal vocalizations serve many important functions, including mate attraction and recognition of individuals [21]. Understanding circadian rhythms in vocal behaviour can offer valuable insights into the function of acoustic signals. For many avian species, alterations in calls throughout the day and in different seasons can reflect the species’ feeding, territorial and reproductive behaviours [22]. Additionally, environmental factors play a role in influencing species-specific calling patterns. For example, many avian species tend to vocalize during daylight hours, particularly at dawn when atmospheric disturbances are thought to be minimized, an adaptation that may optimize the transmission of acoustic signals [23]. According to Gil & Llusia [24], singing at dawn involves relatively low energetic costs, likely because it does not interfere with feeding. Additionally, it is optimal for influencing female mating and establishing territory boundaries. Some bird species are known to exhibit nocturnal calling behaviour, yet there is limited information regarding the patterns and functions of nighttime vocalizations [25]. Throughout the year, birds showcase variation in temporal patterns of vocalizing, with many passerines calling more during the breeding season. Research conducted on the calling behaviour of three warbler species*—Phylloscopus soror, Horornis fortipes* and *Pomatorhinus ruficolli* in China—reveals that they vocalize across three, six and nine months of the year, respectively [26]. Consequently, it is evident that birds’ calling behaviour is subject to change based on the time of day and the season. Investigating avian calling behaviour not only enhances our understanding of their behaviour and of the evolutionary forces that shape this behaviour, but it also forms a crucial foundation for developing effective research and monitoring programmes [26].

The crested argus (*Rheinardia ocellata*) is a rare species that is endemic to Vietnam and Laos and it is classified as critically endangered on the IUCN Red List [27]. Worldwide, the population of the crested argus has declined significantly, with an estimated number of individuals remaining between 10 000 and 20 000, distributed only in Vietnam and Laos [27]. The most serious threat to the species is hunting [28] and habitat loss. In Vietnam, the crested argus occurs in 24 important biodiversity and bird areas [27]. The crested argus has not been recorded since the 2000s in several sites, especially in the lowland areas owing to a sharp increase in trapping [29,30]. Therefore, monitoring and conservation of the crested argus are critical to prevent the decline of this endangered bird species in Vietnam.

The crested argus is very rare and sensitive to human presence; therefore, surveying using visual observations might not be possible. Monitoring the species via their calls is commonly used for this species. Initially, surveys of the crested argus (including the Malaysian crested argus) were done primarily by human surveyors [31–33]. However, surveys using human surveyors can be expensive and limited in space and time [34], and the survey results might be subjective and depend on the surveyors’ experience [35]. This limitation is more obvious when the studied species are rare and distributed in remote areas [36,37]. Therefore, PAM has become a potential method for surveying species that emit typical and loud sounds, including crested argus [38–41]. The use of PAM allows for covering spatial and temporal scales that are almost impossible with traditional human survey methods and is also suitable for tropical forests where visibility is more limited than in other areas [42].

In order to support the monitoring and conservation of the crested argus, understanding the species’ calling behaviour is of great significance. Great argus (*Argusianus argus*)—a species in the same family (Phasianidae) as the crested argus—has two types of calls: short calls and long calls. The species calls throughout the day, with patterns of calling varying depending on the call type, and a substantial portion of calls (approx. 20%) occurring at night [39,43]. Vu Van Tran [43] qualitatively stated that the crested argus often sings a lot between the hours of 5.00 and 9.00 am, while in other studies, the calling behaviour of this species is not mentioned. Our current understanding of the vocal variation of the species concerning the time of day and the seasons is still limited, making it challenging to guide monitoring activities effectively. Therefore, this study aims to provide insights into the circadian rhythms and ecological predictors of crested argus vocal behaviour.

Here, we provide a test of the automated detection of crested argus calls and also investigate environmental predictors of their calling. Our specific aims include the following: (i) investigate the performance of BirdNET transfer learning for automated detection of crested argus calls; (ii) test how varying numbers of training samples influence BirdNET performance; (iii) investigate the predictors of crested argus calling during full 24 h periods and at night; and (iv) provide a qualitative summary of seasonal patterns of calling behaviour. We predicted that, similar to previous reports in great argus (e.g. [39]), we would see (i) a relationship between calling behaviour and lunar phase; (ii) a negative relationship between calling events and rainfall; and (iii) a peak of calling at dawn and dusk. The findings of our study will contribute to guiding the monitoring activities for this species.

## 2. Methods

### (a) Study area

**Figure 1.**
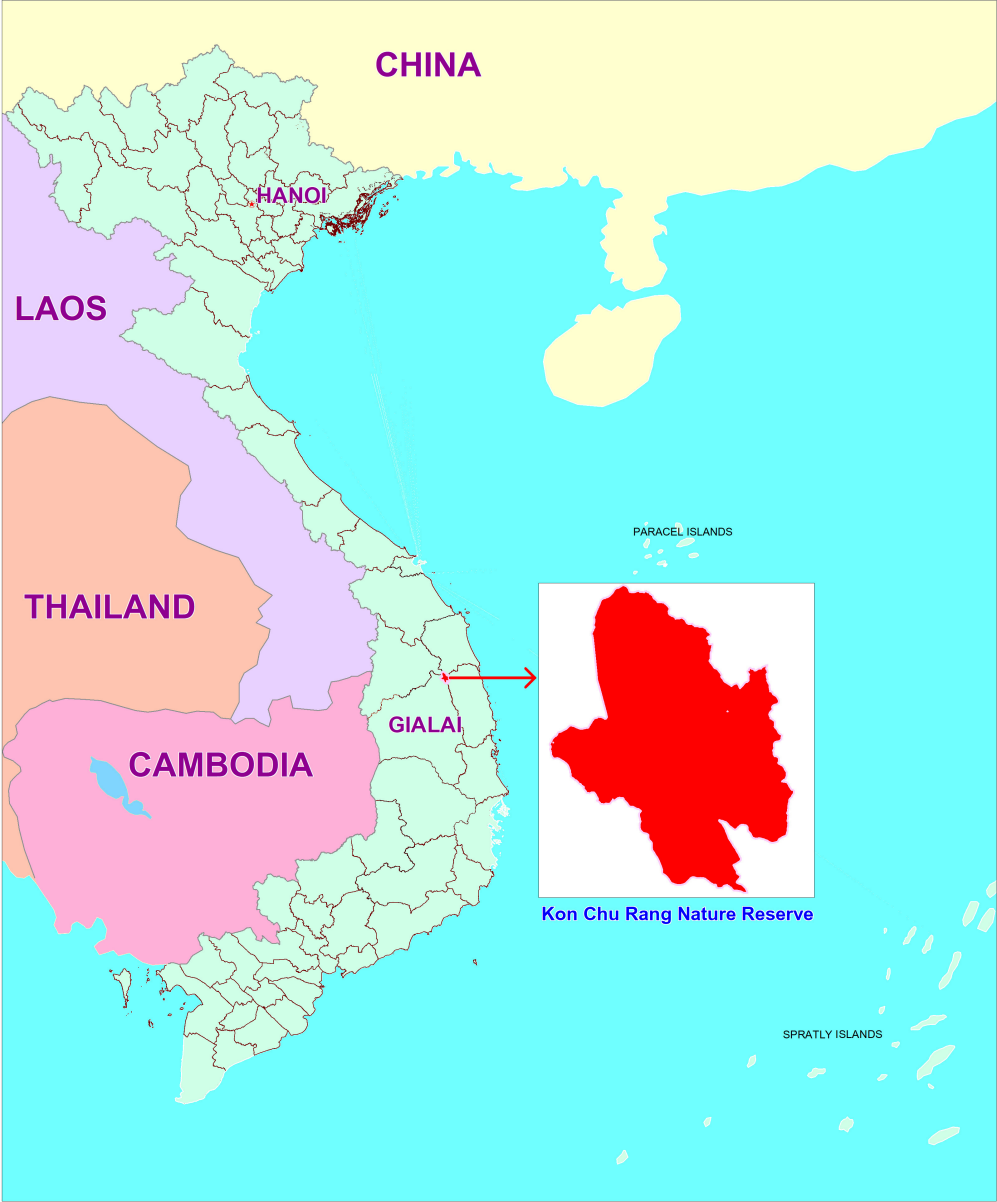
Location of Kon Chu Rang Nature Reserve.

Kon Chu Rang Nature Reserve (NR) is located in KBang district, Gia Lai province (figure 1). The NR was established in 2004 with a total area of 15526.05 hectares, of which 98% is natural forest [44] .

### (b) Study object

#### (i) Recording the crested argus’s calls

The crested argus has loud and resonant calls that can be detected by humans over long distances, at least 500 m. We used mobile smartphones installed with specialized recording software RecForge II – Audio Recorder (Dje073), to record the species' calls. This device has proven effectiveness in recording the calls of the crested argus [45]. Each phone was equipped with a wired BOYA microphone to enhance recording quality and a backup charger to ensure continuous recording for many days. The recorders were attached to tree trunks at about 1−4 m high and set up to record 24 h d^−1^. All smartphones, microphones and backup chargers were wrapped in plastic bags to protect from damage and rainwater. The microphone head was exposed to the outside to ensure the best recording quality. After recording, audio data were saved to disc in Waveform audio file (.wav) format with a sampling rate of 16 000 Hz and resolution of 16 bits. File size for 1 h recording is about 106 Mb. We programmed the recorders to record all day and utilized the default recording level and gain for each recording. Batteries and memory cards were changed when moving the recorders betweeen areas.

To investigate the environmental predictors of crested argus calls, 40 recording posts were set up in 2021 and 30 posts were recorded again in 2023. Recording posts were designed in a 2 × 2 km^2^ grid to ensure independent recording results. We recorded the calls from March to June, as our previous experience indicates that the species is vocally active during this period. Additionally, we lack extensive knowledge of their vocal behaviour at other times of the year. At each point, we conducted recording for 4−5 days continuously. To determine the calling propensity of the crested argus throughout the year, four fixed points were set up in the area where the species calls are detected most frequently in the NR. At these points, we conducted recordings every two months, including six different times of the year (January–February, March–April, May–June, July–August, September–October and November–December). Each point was recorded for up to four continuous days. The coordinates of the recorders, date and type of equipment used were similar between recording occasions through the year.

### (c) Manual data analysis

We used RAVEN Pro 1.6 software (K. Lisa Yang Center for Conservation Bioacoustics, Cornell Lab of Ornithology, USA) for manual analysis of recorded sounds. Crested argus calls were identified in the spectrograms based on the species’ typical sound. Spectrograms were made using the default settings in Raven, with a 512 point (32 ms) Hann window (3 dB bandwidth = 44.9 Hz), with 50% overlap and a 512 point discrete Fourier transform, yielding time and frequency measurement precision of 16 ms and 31.3 Hz.

**Figure 2.**
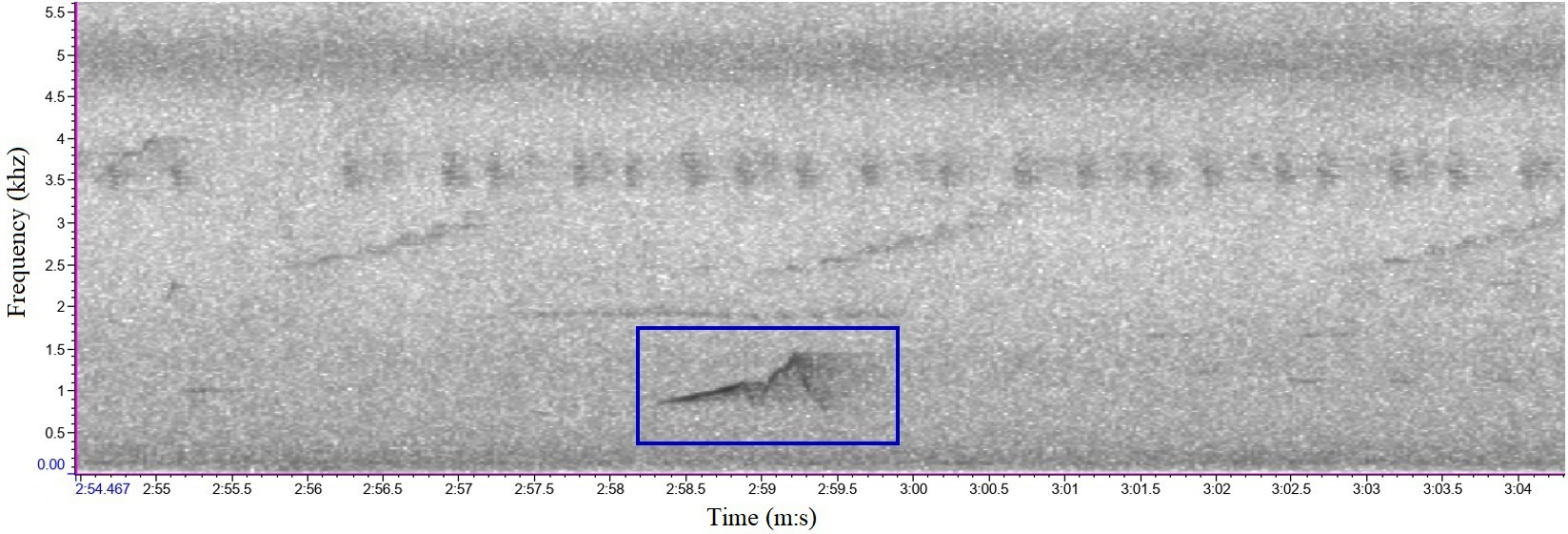
Sound spectrum of the short call of crested argus in Kon Chu Rang Nature Reserve.

**Figure 3.**
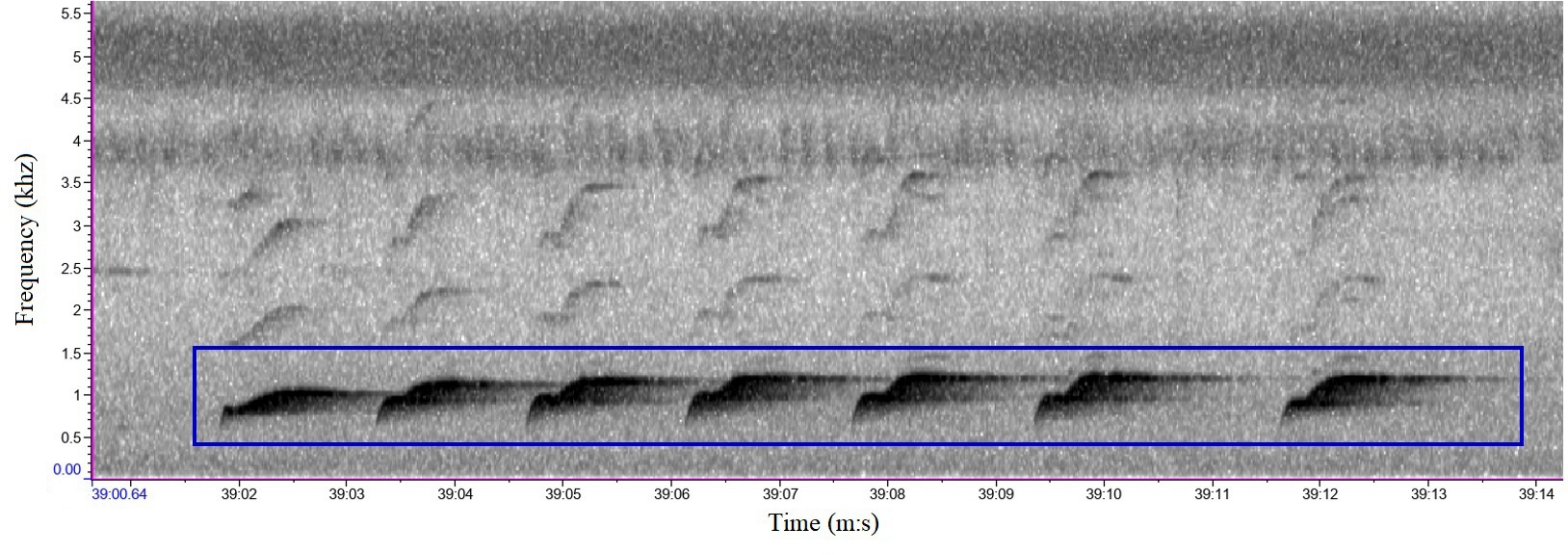
Sound spectrum of the long call of crested argus in Kon Chu Rang Nature Reserve.

We referred to sample sounds from Xeno-Canto (https://xeno-canto.org/) for comparison. In Kon Chu Rang NR, the crested argus have two types of calls: short call with one note and long call with many notes (figures 2 and 3), an average of about six to seven notes [45]. Based on aural and visual cues, the crested argus calls could be easily annotated in the spectrogram. We also listened carefully to the unclear sound segments to confirm the accurate identification of the crested argus calls.

**Figure 4.**
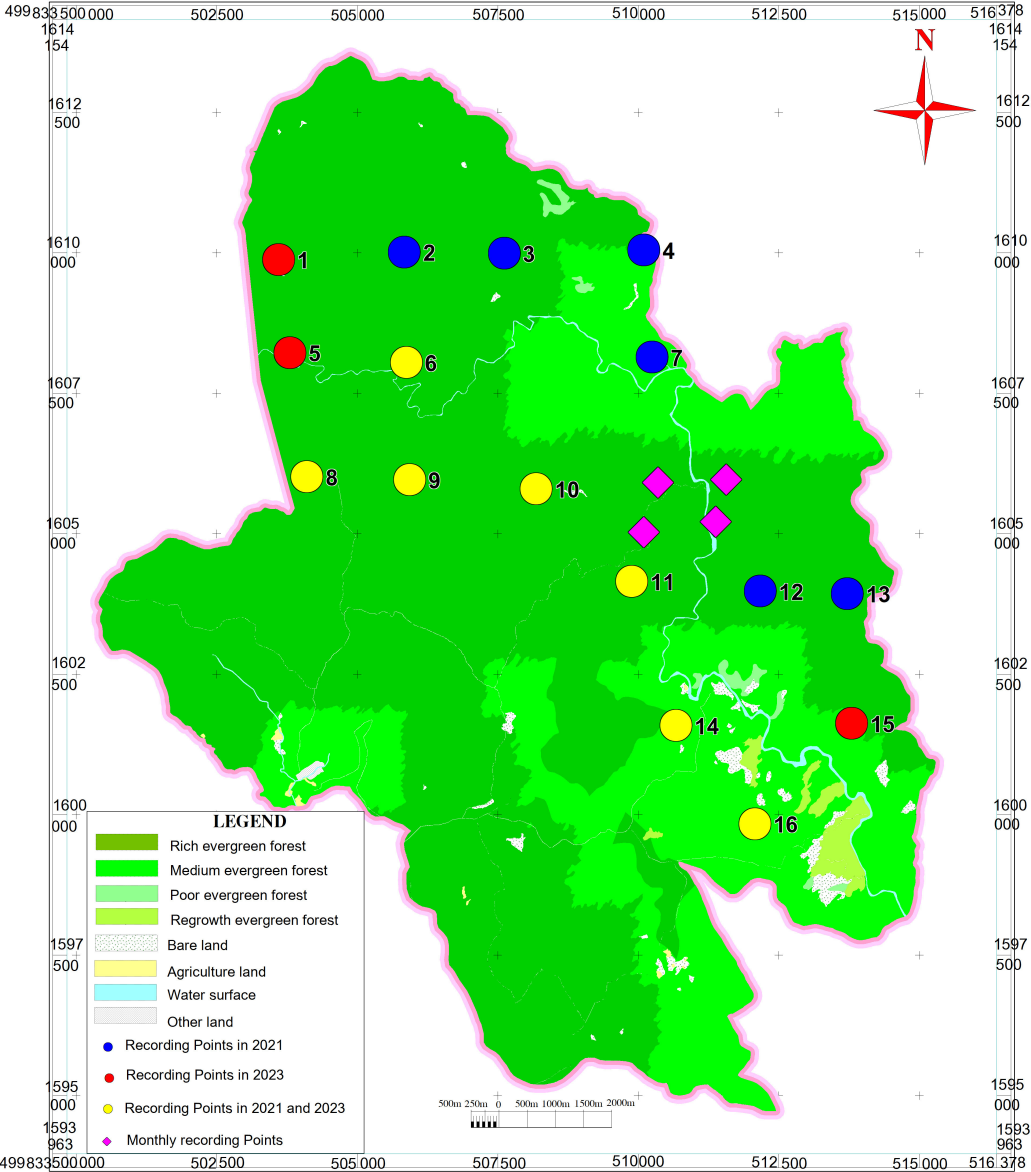
Diagram of recording sites for pheasant calls in Kon Chu Rang Nature Reserve.

All sound files were analysed for the entire day (24 h) to screen for crested argus calls. We then selected the points where the crested argus called with high frequency to count the number of calls per 2 h interval. We used the recording for 36 days in 2021 and 2023 for the sounds analysis to determine the calling frequency variation in a day (table 1, figure 4). In addition, the numbers of crested argus calls during the day were also analysed for each call type.

**Table 1.** Spectrogram screening time for crested Argus in Kon Chu Rang Nature Reserve.

no. recording point	geographical coordinates		spectrogram screening time (local time)	
	latitude	longitude	2021	2023
1	14.481.995	108.629.804		0–24 h (08/5/2023)
2	14.503.290	108.614.731	0 h (28/4) to 24 h (29/4/2021)	
3	14.481.666	108.600.795	0 h (28/4) to 24 h (29/4/2021)	0–24 h (13/5/2023)
4	14.465.833	108.613.821	0 h (29/4) to 24 h (30/4/2021)	0–24 h (08/5/2023)
5	14.504.854	108.593.522	0 h (04/5) to 24 h (05/5/2021)	0–24 h (23/5/2023)
6	14.502.866	108.629.111	0 h (04/5) to 24 h (05/5/2021)	
7	14.521.708	108.539.899	0 h (17/5) to 24 h (18/5/2021)	0–24 h (18/5/2023)
8	14.521.235	108.556.888	0 h (17/5) to 24 h (18/5/2021)	0–24 h (18/5/2023)
9	14.519.747	108.577.737	0 h (18/5) to 24 h (19/5/2021)	0–24 h (18/5/2023)
10	14.540.936	108.596.887	0 h (28/5) to 24 h (29/5/2021)	
11	14.558.164	108.595.531	0 h (28/5) to 24 h (29/5/2021)	
12	14.557.657	108.572.516	0 h (30/5) to 24 h (01/6/2021)	
13	14.557.870	108.556.007	0 h (06/6) to 4 h (07/6/2021)	
14	14.540.099	108.556.327	0 h (06/6) to 24 h (07/6/2021)	0–24 h (02/6/2023)
15	14.541.667	108.537.119		0–24 h (03/6/2023)
16	14.556.653	108.535.238		0–24 h (02/6/2023)

### (d) Automated detection of crested argus calls

We were interested in evaluating the performance of an automated detection approach, and we focused on using BirdNET [10]. The current version of BirdNET (v. 2.4) has been trained on over 6000 different species; however, the crested argus was not included in the training dataset of the current version. Recent advances in transfer learning using BirdNET [17] mean that we could use the feature embeddings from BirdNET to train a new classifier for crested argus. We used 16 h of recordings that contained crested argus calls for training, 8 h for validation and 8 h for testing to report our final results. To ensure machine learning best practices, training, validation and test recordings were all taken from different recording days. The numbers of calls used for training varied (see below), and the recordings for validation contained 41 calls, while the recordings for final testing contained 22 calls.

We used two separate approaches to create training datasets. First, we used all the manual annotations as outlined above for the ‘crested argus’ class. To create a ‘noise’ class, we used a band-limited energy detector (BLED) focusing on the 500–1800 Hz frequency range implemented using the ‘gibbonR’ v. 1.0.1 R package [46]. Briefly, to use the BLED detector, we created a spectrogram of the recording with zero overlap and a window size = 512, and we then calculated the energy in each time window of the spectrogram. We then used the ‘quantile’ function in the ‘stats’ R package in base R v. 4.4.0 [47] to determine the 0.15 quantile of the distribution; we considered anything at this value or lower to be ‘noise’ and anything above it to be ‘signal’. We then pooled the time windows and considered anything between 3 s and 12 s duration to be a candidate signal. We used the start and stop times of the crested argus annotations to identify parts in the recording that did not contain crested argus calls and ran the BLED detector over these segments of the recordings. This approach resulted in 104 crested argus samples and 108 noise samples. We found that this training dataset led to suboptimal performance (see §3), so we tested a different approach to create training data. For this, we used the BLED detector as outlined above; however, we ignored the human annotations. A single observer (D.J.C.) then manually sorted the detections into two categories: ‘crested argus’ and ‘noise’. This resulted in 31 crested argus samples and 390 noise samples; we used this training dataset for randomization experiments described below. This approach effectively ignored many of the low signal-to-noise ratio calls, leading to a dataset of only high-quality crested argus calls.

We were interested to see how the number of training samples would influence the performance of our models, so we randomly selected 5, 10, 15, 20, 25 and 30 crested argus and noise samples over five iterations. We then used the ‘train.py’ function implemented in BirdNET (https://github.com/kahst/BirdNET-Analyzer) for each of our training datasets. This function allows users to train a new classifier for classes that are not currently in BirdNET using BirdNET embeddings. We used the default settings apart from setting fmin = 500 Hz and fmax = 1800 Hz. BirdNET also allows for data augmentation in the training process. We compared the results of BirdNET trained only on high-quality calls with the results when the calls were divided into 3 s segments, as the default settings take the centre 3 s for each training clip. We also used the ‘mixup’ option, which is a data augmentation technique that synthesizes a new sample by combining two existing samples [48]. We used the default mixup settings, wherein augmentation_ratio = 0.25 and alpha = 0.2, and also manually changed the settings where augmentation_ratio = 0.75 and alpha = 1.0. The augmentation ratio specifies the percentage of samples that are modified, and alpha controls the shape of the beta distribution, which is used to generate the mixing ratio for the two samples; a higher alpha means more uniform mixing of the two samples.

We then deployed the newly trained classifiers over the validation dataset and reported F1, precision, recall using the ‘caret’ v. 6.0-94 package [49] and area under the receiver-operating (AUC-ROC) curve calculated using the ‘pROC’ v. 1.18.5 package [50]. The current version of BirdNET was trained on great argus (*A. argus*), so we also tested the performance on our crested argus data. However, the performance was poor (all metrics = 0), so we did not move forward with this analysis. BirdNET presently returns an associated class and confidence score for each 3 s window in a longer recording, so we considered any BirdNET detection within a 3 s window before or after the manual annotation to count as a true positive detection. Once we determined the best-performing model configuration, we deployed that model over our test dataset and calculated the performance metrics as described above.

To further explore model performance on our validation and test datasets, we used logistic regression to convert BirdNET confidence scores to probabilities [51]. We used the BirdNET graphical user interface v. 1.4.0 and the segments review tab to manually sort 100 3 s BirdNET detections from our test dataset into true and false positives. We then fit a logistic regression using the ‘bbmle’ package [52] to relate the binary outcome (true or false positive) to the BirdNET confidence score as the predictor. The logistic regression model can then be used to determine which BirdNET confidence scores are associated with a certain probability of a true detection. We compared this model with BirdNET confidence score as a predictor to a null model that simply had the binary outcome variable using small-sample corrected Akaike information criterion (AICctab) in the ‘bbmle’ v.1.0.25.1 R package [52].

### (e) Modelling the predictors of crested argus calling

For all of the recording hours in our dataset (*n* = 891), we indicated whether crested argus were manually detected during that time period or not. We used the ‘lunar’ v. 0.2-01 R package [53] to determine lunar phase and the ‘Nasapower’ v. 4.2.1 R package [54] to get hourly rain data that correspond with our study period. For the rain data, we input the mean latitude and longitude for all recorders, and the resolution is 55.5 km × 55.5 km cells, which means we were not able to capture micro-habitat variation. We created a series of generalized linear mixed models using the R package ‘glmmTMB’ v. 1.1.9 [55]. Our first set of models considered the entire 24 h period. We assigned each hour to a time category as follows: sunrise (05:00 LT), morning (06:00–11:00 LT), afternoon (12:00–17:00 LT), sunset (18:00 LT), evening (19:00–23:59 LT) and late night (00:00–04:00 LT). The first model was a null model that included a binary (0 or 1) indicator of the presence or absence of argus calls in a particular hour as the outcome, with the site and date as nested random effects. The second model contained rainfall as a predictor, the third model contained time category as a predictor and the last model contained both rainfall and time category as predictors. We compared model fit using AICctab implemented in the ‘bbmle’ v. 1.0.25.1 R package [52] and used the ‘performance’ v. 0.12.0 package [56] to assess normality of model residuals. For the second set of models, we focused only on calls that occurred at night between 18:00 and 05:00 (inclusive). The first model was the null model and only contained site and date as nested random effects. The second model contained lunar phase (either New, Waxing, Full, or Waning) and the third model contained the proportion of lunar illumination as a predictor. We compared the three models using AIC as described above. We used the ‘MuMin’ v. 1.47.5 R package [57] to calculate a pseudo-*R* ^2^ value.

### (f) Data availability

All data needed to recreate the analyses are available on GitHub: https://github.com/DenaJGibbon/crested-argus.

## 3. Results

### (a) Qualitative analysis of calling behaviour

Using data from the manual annotations of spectrograms, we found that the crested argus called mainly during two periods: from 4 am to 1 pm and from 4 pm to 9 pm (table 2). No calls were recorded at other times of the day.

**Table 2.** Average calling rate of the crested Argus during different hours of the day.

TT	time period	no. of statistical days	total number of calls	calling rate (times/recording point)	ratio (%)
1	00–01 h	36	0	0.00	0.00
2	01–02 h	36	0	0.00	0.00
3	02–03 h	36	0	0.00	0.00
4	03–04 h	36	0	0.00	0.00
5	04–05 h	36	3	0.08	0.81
6	05–06 h	36	47	1.31	12.70
7	06–07 h	36	88	2.44	23,78
8	07–08 h	36	52	1.44	14.05
9	08–09 h	36	34	0.94	9.19
10	09–10 h	36	21	0.58	5.68
11	10–11 h	36	2	0.06	0.54
12	11–12 h	36	4	0.11	1.08
13	12–13 h	36	2	0.06	0.54
14	13–14 h	36	0	0.00	0.00
15	14–15 h	36	0	0.00	0.00
16	15–16 h	36	1	0.03	0.27
17	16–17 h	36	8	0.22	2.16
18	17–18 h	36	9	0.25	2.43
19	18–19 h	36	61	1.69	16.49
20	19–20 h	36	30	0.83	8.11
21	20–21 h	36	8	0.22	2.16
22	21–22 h	36	0	0.00	0.00
23	22–23 h	36	0	0.00	0.00
24	23–24 h	36	0	0.00	0.00

**Figure 5.**
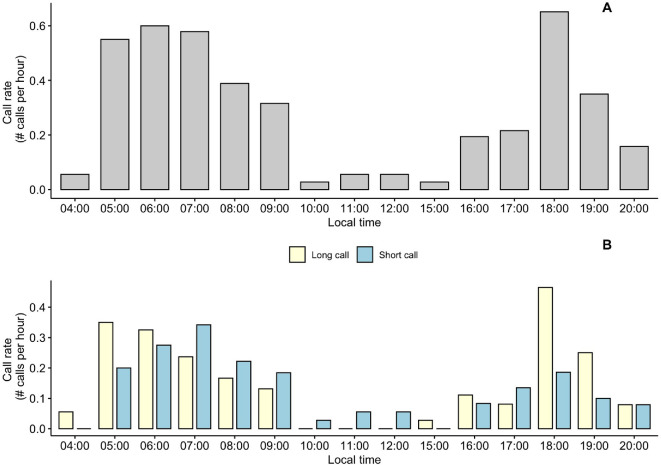
Number of crested argus calls per hour, adjusted for the total number of recording hours. (A) The call rate per hour for all crested argus calls and (B) the call rate per hour by call type.

Crested argus start to call at 4:00 am with a low rate (average 0.08 times h^−1^), increasing sharply between 5:00 and 7:00 am, reaching maximum rate between 6:00 and 7:00 am (an average of 2.44 times h^−1^), then the frequency of calling began to decrease rapidly. At noon (from 10 am to 1 pm), the crested argus called very little. In the afternoon, the crested argus started calling from 4:00 pm and the frequency of calling began to increase sharply from 6:00 pm to 7:00 pm, reaching its maximum frequency during this period with an average frequency of 1.69 times h^−1^. After this time, the frequency of calling decreased sharply and crested argus stopped calling after 9:00 pm (figure 5).

If dividing the day into two sessions: morning (from 0:00 to 12:00 am) and afternoon (from 12 am to 12 pm), the crested argus called more in the morning, accounting for 67.8% of the calls of the day. In the morning, the crested argus tend to use short calls, while in the afternoon, long calls were used more frequently.

The calling frequency of the crested argus increased gradually from January to April, during which the crested argus called the most from March to April with an average frequency of 31.88 times d^−1^ (table 3). After that, the calling frequency decreased steadily from May to August. From September to January in the next year, the calling frequency of the species is very low. In general, the crested argus called most from March to June; the number of calls recorded during this time accounts for 80% of the number of calls recorded throughout the year at recording sites.

**Table 3.** Average calling rate of crested Argus at different times of the year.

no	time in year	average calling rate (times/day) at recording points				average calling rate (times/day/recording point)
		point 1	point 2	point 3	point 4	
1	Jan–Feb	5.75	4.5	4.5	4.25	4.75
2	Mar–Apr	35.75	18	29.25	44.5	31.88
3	May–Jun	23	11.25	26.25	18.25	19.69
4	Jul–Aug	7.5	4.75	8	8.75	7.25
5	Sep–Oct	0	0	0.5	1.5	0.50
6	Nov–Dec	0.25	0	0	0	0.06

### (b) Automated detection results

**Figure 6.**
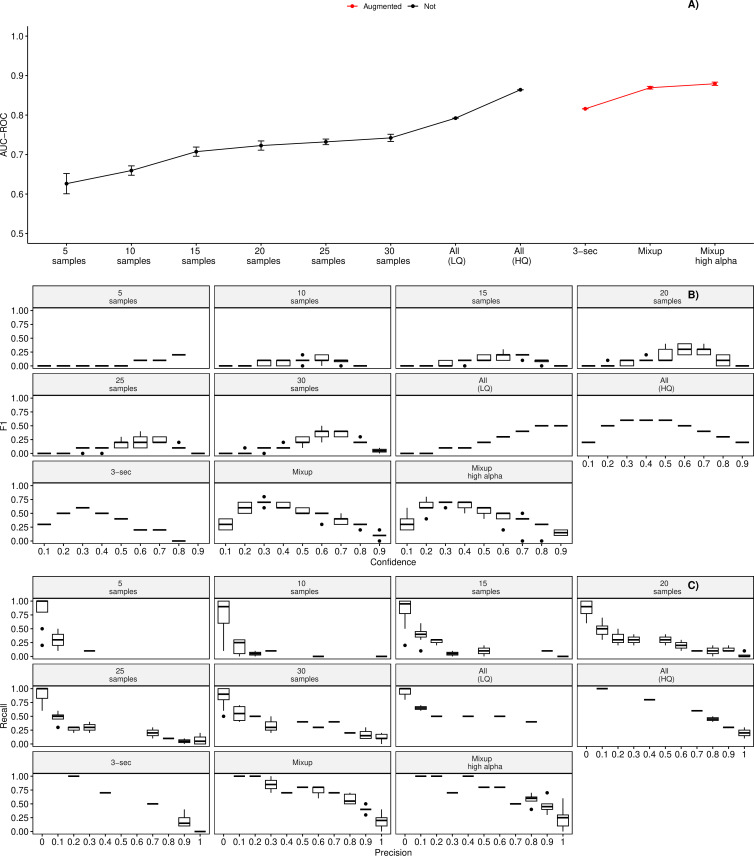
Boxplots showing the AUC (A), F1 as a function of confidence score (B), and precision versus recall (C) for all training data subsets on the validation dataset. For training samples from 5 to 30, we randomly selected the specified number of samples for the ‘crested argus’ and ‘noise’ category. For all samples (LQ), this training dataset was created using all manual annotations, and for all samples (HQ), this training dataset was created using high-quality sorted detections from the BLED detector. For the three data augmentation approaches, only the high-quality calls were used. Based on AUC and maximum F1 score, we determined that the model trained on all the high-quality crested argus and noise samples with mixup at a high alpha had the best performance.

Using the validation set, we found that the training dataset that contained training samples detected using the BLED detector and then subsequently sorted manually led to substantially better performance than using manual annotations, even though the number of positive samples was smaller (31 versus 104 crested argus samples). See figure 6 for a comparison of F1, precision, recall and AUC for different training datasets. We report the final performance on a test dataset that was not used for initial training or validation and found that for the model trained on all the high-quality training samples and all the noise samples without data augmentation, the best F1 score was at a confidence level of 0.4, F1 score = 0.70, precision = 0.72, recall = 0.67 and AUC–ROC = 0.79. For the model with data augmentation (mixup with high alpha), the best F1 score was at a confidence level of 0.3, F1 score = 0.63, precision = 0.57, recall = 0.71 and AUC–ROC = 0.85.

### (c) Confidence scores as probabilities

**Figure 7.**
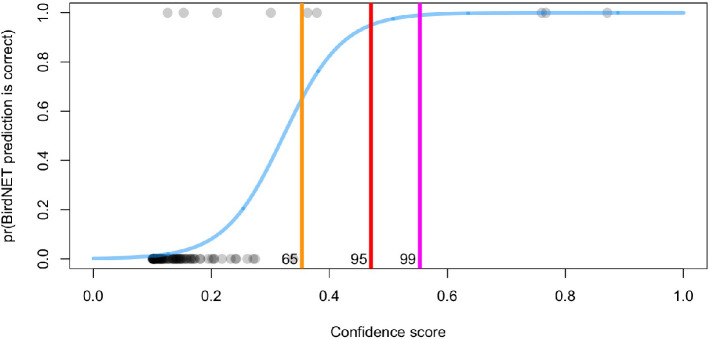
The probability of a correct BirdNET prediction for crested argus on the test dataset (validated predictions shown in grey) increased with prediction score. The blue line indicates the logistic regression line, and the yellow, red and pink lines indicate confidence scores that have a 65%, 95% and 99% chance of being correct, respectively.

We manually sorted 100 3 s BirdNET detections (model using mixup with high alpha) from our test dataset. We fit a logistic regression model with the binary outcome (true or false positive) and BirdNET confidence score as the predictor. We found that there was strong support for a relationship between BirdNET confidence score and the probability that the prediction was correct (figure 7) and this model received 100% of the model weight over the null model using AIC (delta AIC = 26.9). At a confidence score of 0.55, the prediction had a 95% probability of being correct.

### (d) Modelling predictors of crested argus calling

**Figure 8.**
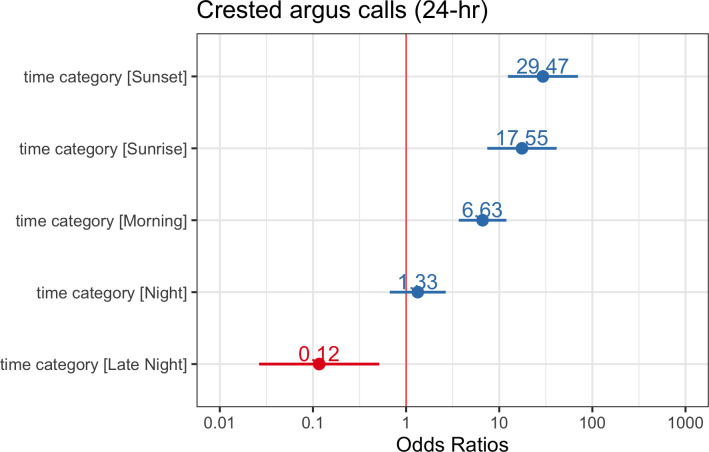
Odds ratios with a 95% confidence interval for crested argus calling over a 24 h period. If odds ratio = 1 then this predictor does not impact the outcome, whereas odds ratio >1 means higher odds of outcome. We considered predictors with confidence intervals that do not cross 1 to have an impact on crested argus calling. In the plot above, the afternoon time category is the reference category.

**Figure 9.**
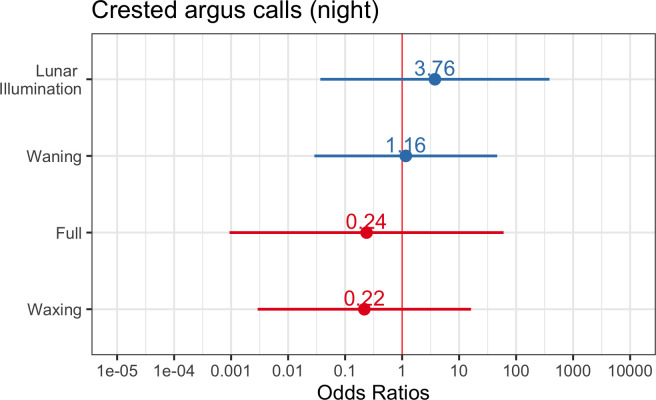
Odds ratios with a 95% confidence interval for crested argus calling at night. If odds ratio = 1, then this predictor does not impact the outcome, whereas odds ratio >1 means higher odds of outcome. None of the above predictors had an impact on crested argus night-time calling. In the plot above, the ‘new moon’ category is the reference category.

For our model selection using AIC for all calls during a 24 h period, we found that the top model included time category as a predictor. This top model comprised >99% of the model weight when doing model comparison with AIC; the top model performed substantially better than the intercept-only model (ΔAIC = 184.7; <0.001% of model weight) and the pseudo-*R* ^2^ value indicated that the predictor variables explained approximately 30% of the variance and the entire model (predictors and random effects) explained approximately 50% of the variance. See figure 8 for a plot of the odds ratio for the top model of crested argus calling over a 24 h period. For calls that only occur at night, we found that our intercept-only model was ranked highest using AIC, indicating that lunar phase and lunar illumination were not reliable predictors of crested argus calling in this dataset (figure 9).

## 4. Discussion

Here, we provide a qualitative and quantitative examination of crested argus vocal behaviour. We found peak calling behaviour at sunrise and sunset but did not find any relationship between rainfall, lunar phase or lunar illumination. It is possible that the environmental predictors we used were not at a sufficiently high resolution, and future work should incorporate local measures of rainfall and lunar illumination. For the automated detection approach, we found that including 31 high-quality training samples, along with 390 noise samples, led to higher performance. We assume that the removal of the low-quality crested argus calls led to this improved performance, as we compared both low- and high-quality training data with the same number of noise samples. However, we did not find evidence that transfer learning with a small number of samples led to acceptable performance in this context, as both F1 and AUC scores remained low. We also found that this system handles unbalanced training data and in fact performed better with a higher number of noise samples.

We found that using data augmentation led to an AUC-ROC of 0.85 on the test dataset and that there were very few false positives. We were able to determine a BirdNET confidence score wherein there was a 95% probability that the detection was true. This level of performance may be satisfactory for occupancy modelling, wherein lower recall but higher precision are generally required [58]. However, we caution that our test dataset was relatively small and might not be representative of a landscape-scale study. Therefore, we suggest future applications for crested argus evaluate BirdNET site-level performance before deploying over a large-scale dataset; this is true for all signals and applications.

It is also possible to use detectors such as BirdNET in an active learning approach [59], wherein the detector is used in conjunction with human validation to create a larger training dataset. However, even if precision is relatively low, manual sorting of detections into true and false positives is often less time-consuming than manually annotating spectrograms of long-term recordings. Our manual sorting of true and false positives showed that at high confidence scores there were very few false positives, but at lower confidence scores, the false positives were often other species of birds or insect noise. Future work benchmarking BirdNET against other state-of-the-art models for this species, such as Perch [60], will also be informative.

The calling behaviour of the crested argus during the day is consistent with the behaviour of birds in general and species in the Phasianidae family in particular. If considered only in the morning, this result is quite similar to the study on the calling behaviour of the crested argus in Ngoc Linh Nature Reserve [41] and great argus [39]. In the morning, a higher proportion of short calls were recorded than long calls, while in the afternoon, long calls were used more than short calls. In great argus, the short calls are thought to serve a mate attraction purpose, and long calls a territorial purpose [61]. However, it is unclear whether the behaviour context of calling is the same for crested argus and great argus. Unlike great argus on Malaysia Borneo [39], we did not find a substantial amount of crested argus calls at night. The authors propose that the high proportion of night calls in great argus on Borneo, which contrasts with reports of them being strictly diurnal on Sumatra [62], may be related to differences in predation pressure, as Borneo lacks tigers that are found on Sumatra. Although tigers are functionally extinct in Vietnam, it is possible that tigers shaped the evolutionary landscape of crested argus.

The study on crested argus in Kon Chu Rang NR is the first study in Vietnam in which the calling frequency of the species is determined for the whole day. This result has great significance in orienting research and monitoring activities on the crested argus. To our knowledge, this also is the first study to qualitatively assess the variation of the calling rate of crested argus over the years. Crested argus often call more during the breeding season, and it seems likely that the calls are emitted by males to attract females. The time of year when the crested argus calls a lot (March–June) may be the main time in the breeding season of the species. In particular, in this study, a female crested argus recorded with its offspring by camera trap in June 2022 confirmed this statement. Findings on the calling behaviour variation during the year also provide a scientific basis to guide research and monitoring activities of the crested argus.

The best time for a PAM study focusing on only crested argus could feasibly schedule recording for only 5:00−9:00 and 18:00−19:00, and the best season for monitoring the species is from March to June. We suggested that automated detection can be potentially useful in detecting the calls of crested argus, especially in projects with much larger datasets. Nation-wide monitoring programmes, not only for crested argus but also for other vocal galliform species, can be developed for the species and data can be sent to a specialized centre for analysis using automated detection.

## Data Availability

Data needed to recreate the analyses are available on GitHub: [63]
